# Importance of epicardial adipose tissue localization using cardiac magnetic resonance imaging in patients with heart failure with mid‐range and preserved ejection fraction

**DOI:** 10.1002/clc.23644

**Published:** 2021-06-04

**Authors:** Gijs van Woerden, Dirk J. van Veldhuisen, Thomas M. Gorter, Vanessa P. M. van Empel, Martin E. W. Hemels, Eric J. Hazebroek, Sophie L. van Veldhuisen, Tineke P. Willems, Michiel Rienstra, Berend Daan Westenbrink

**Affiliations:** ^1^ Department of Cardiology University of Groningen, University Medical Center Groningen Groningen Netherlands; ^2^ Department of Cardiology University of Maastricht, Medical University Centre Maastricht Maastricht Netherlands; ^3^ Department of Cardiology Rijnstate Hospital Arnhem Netherlands; ^4^ Department of Surgery Rijnstate Hospital Arnhem Netherlands; ^5^ Department of Radiology University of Groningen, University Medical Center Groningen Groningen Netherlands

**Keywords:** cardiac magnetic resonance imaging, EAT, HFmrEF, HFpEF

## Abstract

**Background:**

Epicardial adipose tissue (EAT) has been implicated in the pathophysiology of heart failure (HF) with left ventricular ejection fraction (LVEF) >40%, but whether this is due to a regional or global effect of EAT remains unclear.

**Hypothesis:**

Regional EAT is associated with alterations in local cardiac structure and function.

**Methods:**

Patients with HF and LVEF >40% were studied. Cardiac Magnetic Resonance imaging was used to localize EAT surrounding the right ventricle (RV) and LV separately, using anterior‐ and posterior interventricular grooves as boundaries. Atrial‐ and ventricular EAT were differentiated using the mitral‐valve position. All EAT depots were related to the adjacent myocardial structure.

**Results:**

102 consecutive HF patients were enrolled. The majority of EAT was present around the RV (42% of total EAT, *p* < .001). RV‐EAT showed a strong association with increased RV mass (*β* = 0.60, *p* < .001) and remained associated with RV mass after adjusting for total EAT, sex, N‐terminal prohormone of brain natriuretic peptide (NT‐proBNP), renal function and blood glucose. LV‐EAT showed a similar association with LV mass in univariable analysis, albeit less pronounced (*β* = 0.24, *p* = .02). Atrial EAT was increased in patients with atrial fibrillation compared to those without atrial fibrillation (30 vs. 26 ml/m^2^, *p* = .04), whereas ventricular EAT was similar (74 vs. 75 ml/m^2^, *p* = .9).

**Conclusions:**

Regional EAT is strongly associated with local cardiac structure and function in HF patients with LVEF >40%. These data support the hypothesis that regional EAT is involved in the pathophysiology of HF with LVEF >40%.

## 1 INTRODUCTION

Heart failure (HF) with a left ventricular ejection fraction (LVEF) >40% (i.e. HF with mid‐range EF [HFmrEF] and preserved EF [HFpEF]) is an emerging heart disease with poor prognosis for which currently no evidence based therapies exists.^1^, ^2^, ^3^, ^4^ Epicardial adipose tissue (EAT) is increasingly recognized as a potential culprit in the pathophysiology of HF patients with LVEF >40%.^5^, ^6^, ^7^

EAT is a layer of visceral fat that is in direct contact with the heart that has the potential to influence myocardial structure and function. For instance, experimental and clinical studies have also shown that local EAT can affect the electrophysiological properties of the atrial myocardium, resulting in slowed conduction and fractionated electrograms.^8^, ^9^ This is at least partially due to myocardial infiltration of EAT, which disrupts myocardial ultrastructure and promotes fibrosis.^9^ In contrast, the accumulation of EAT has also been associated with increased cardiac filling pressures suggesting that it increases the mechanical load on the myocardium.^6^, ^7^, ^10^

The total EAT volume is markedly increased in HFmrEF and HFpEF patients compared to healthy controls, and is associated with disease severity.^5^, ^7^ However, as these associations were established with measures of global EAT volume, it is unknown if the effects of EAT in this population are explained by regional or global effects of EAT on the myocardium.^9^ We therefore investigated the relationship between local EAT accumulation and adjacent local myocardial structure and function using cardiac magnetic resonance (CMR) imaging in patients with HFmrEF and HFpEF.

## 2 METHODS

### 2.1 Study population

We studied symptomatic HF patients with an LVEF>40% with an N‐terminal prohormone of brain natriuretic peptide (NT‐proBNP) of >125 pg/ml and echocardiographic evidence for LV diastolic dysfunction, left atrial dilatation and/or LV hypertrophy, according to current European Society of Cardiology criteria.^1^ All patients were part of a standard diagnostic protocol for HF patients with an LVEF>40%. This protocol consisted of a thorough examination including laboratory testing, echocardiography and CMR imaging. Patients with congenital heart disease and more than moderate left‐sided valvular disease were excluded.

The Institutional Review Board of the University Medical Center Groningen approved the retrospective study and the need for individual informed consent was waived. The present study was in concordance with the principles outlined in the Declaration of Helsinki. The present analysis was based on a database previously used by our group, and was extended with 38 more cases.^5^ In addition, 82 patients participated in the Ventricular tachyarrhythmia detection by Implantable Loop Recording in Patients with Heart Failure and Preserved Ejection Fraction (VIP‐HF) registry (NCT01989299) to evaluate the incidence of sustained ventricular arrhythmias in patients with HFpEF.^11^

### 2.2 Cardiac magnetic resonance imaging

#### 2.2.1 Imaging protocol

CMR was performed using a standard protocol for the acquisition of cardiac volumes and functional parameters, as previously published.^5^ In brief, all CMR studies were performed using a standard 1.5 Tesla scanner (Philips, Amsterdam, The Netherlands & Siemens, Erlangen, Germany). ECG‐triggered cine loop images were obtained during breath hold at end‐expiration, using a retrospectively gated cine steady‐state, free‐precession sequence. Approximately 15 short‐axis slices from base to apex were obtained, including both atria.

#### 2.2.2 Imaging analyses

Cine loop images were analyzed off‐line by two observers (G.W. and T.M.G.) using dedicated software (QMass 7.6 and 8.1, QStrain 2.0, Medis, Leiden, The Netherlands). Papillary muscles were included when measuring mass, and excluded when measuring volumes.^12^ All volumetric measurements on CMR (including EAT) were indexed for body surface area, using the DuBois method.^13^

**FIGURE 1:**
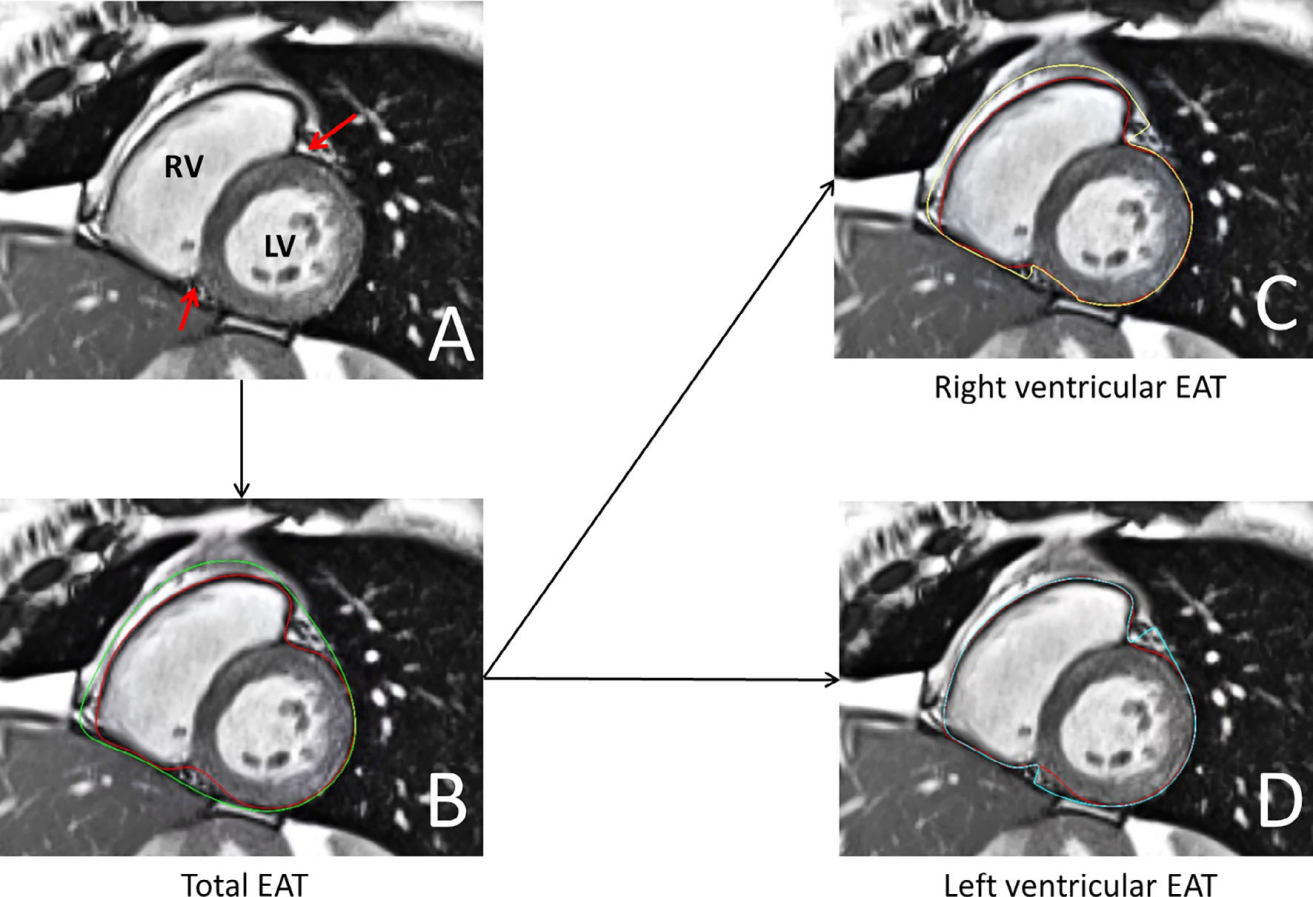
Overview of right‐ and left ventricular epicardial adipose tissue (EAT) measurements on short‐axis stacks. (A) The red arrows indicate the anterior and posterior interventricular grooves which are used as borders. (B) Short axis frame measuring total ventricular EAT, red line marks myocardium, green line marks the pericardium. (C) Measurement of right ventricular EAT. (D) Measurement of left ventricular EAT. LV, left ventricle; RV, right ventricle

EAT is the adipose tissue situated between the outer wall of the myocardium and the visceral layer of the pericardium.^14^ EAT was manually delineated on end‐diastolic short‐axis slices, working from the most basal slice toward the most apical slice.^5^, ^15^ The mitral valve annulus position was used to differentiate between atrial and ventricular EAT. Atrial EAT was defined as the EAT around the atria, located above the mitral valve and below the right pulmonary artery. Ventricular EAT was defined as the EAT around the ventricles, lying between the mitral valve and the ventricular apex.^15^ To distinguish EAT located around the RV from EAT around the LV, the anterior and posterior interventricular grooves on short‐axis slices were used as boundaries (Figure 1). The use of the interventricular grooves as landmarks for distinguishing the RV from the LV has been described earlier in an autopsy study performed by Corradi and colleagues.^16^ EAT volumes were calculated by summation of EAT volume of each slice using the modified Simpson's rule.^17^ In addition, the adipose origins of the proposed EAT were verified by comparing the pre‐ and post‐contrast T1 times of the proposed EAT with T1 times of the subcutaneous fat using T1 mapping at mid ventricular level, as described previously.^5^ If uncertainty occurred regarding the distinction between epicardial adipose tissue and paracardial adipose tissue (adipose tissue outside the pericardial sac), a third independent reviewer (B.D.W.) was consulted.

### 2.3 Statistical analysis

Data are presented as numbers (percentage), mean ± SD or median with interquartile ranges. Differences between groups were analyzed using the independent samples *t*‐test, the Wilcoxon rank test, the Chi‐squared test or the Fisher's exact test where appropriate. Associations between EAT and cardiac structure and function were analyzed using univariable and multivariable linear regression models. In the multivariable regression model, the association between local EAT with myocardial structure was adjusted for the four clinical characteristics that were most strongly associated with total EAT; with forced entry of total EAT. Prior to regression analysis, covariates that were non‐normally distributed were log transformed. As a sensitivity analysis, we also assessed the relationship between EAT and local cardiac structural and functional abnormalities in the normal body mass index (BMI) range (BMI < 25 kg/m^2^). Intraobserver and interobserver variability for EAT and RV mass measurements were assessed using the intraclass correlation coefficient. Statistical analyses were performed using SPSS (Version 23, Chicago, Illinois). Statistical significance was considered achieved at a *p*‐value <.05.

## 3 RESULTS

### 3.1 Patient characteristics

**FIGURE 2:**
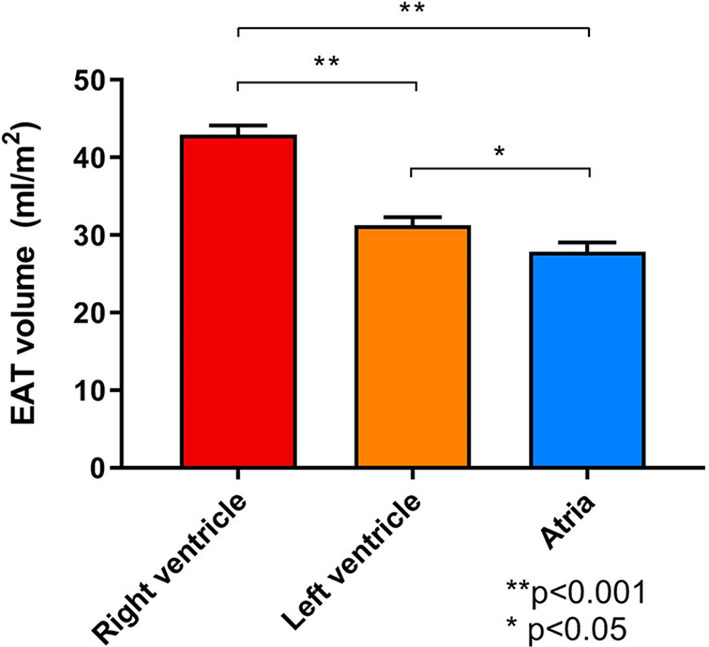
Bar graphs comparing the different local epicardial adipose tissue (EAT) volumes. Volumes are indexed for body surface area. Error bars represent standard error of the mean

**FIGURE 3:**
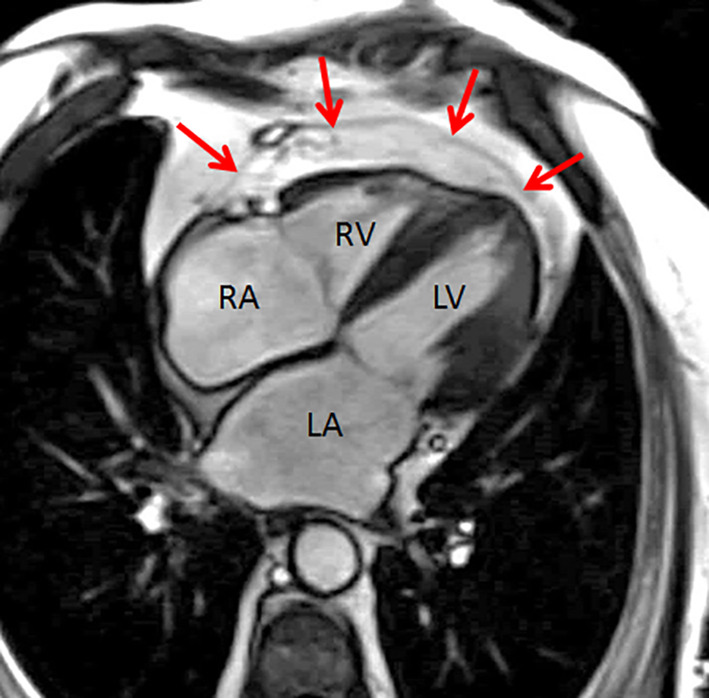
Typical example of epicardial adipose tissue (EAT) distribution around the right ventricle. The red arrows indicate the pericardium. LA, left atrium; LV, left ventricle; RA, right atrium; RV, right ventricle

We studied 102 consecutive HF patients with LVEF >40%. Mean age was 70 ± 10 years, 49% were female and the median NT‐proBNP was 1331 [inter quartile range 683–2531] pg/ml. Other patient characteristics are shown in Table 1. In six HF patients (5.9%), the atria were not sufficiently depicted in the short‐axis stacks, thus the analysis for atrial EAT were based on 96 HF patients. Interobserver variability for cardiac and EAT volumes were excellent: intraclass coefficient all >0.90, *p* < .05. The EAT volume around the RV was significantly higher than the EAT volume around the LV and the atria (43 ml/m^2^ vs. 31 ml/m^2^, vs. 28 ml/m^2^, respectively, all *p* < .05) (Figures 2 and 3). Even when correcting for individual chamber volumes, RV‐EAT remained significantly higher than LV‐EAT and atrial EAT (*p* < .001 and *p* = .004, data not shown).

**TABLE 1:** Patient characteristics

	Heart failure patients
	(*n* = 102)
Age, years	70 ± 10
Sex, female	50 (49)
Body mass index, kg/m^2^	29.5 ± 5.8
**Vital signs**	
Systolic blood pressure, mmHg	142 ± 22
Diastolic blood pressure, mmHg	75 ± 14
Heart rate, bpm	72 ± 13
**Comorbidities, *n* (%)**	
Hypertension	79 (78)
Atrial fibrillation	49 (48)
Myocardial infarction	24 (24)
Diabetes mellitus	40 (39)
COPD	18 (18)
*NYHA class*, *n* (*%*)	
II	59 (58)
III	42 (42)
**Medications, *n* (%)**	
Beta blockers	92 (90)
ACEi/ARB	73 (72)
MRA	41 (40)
Loop diuretics	91 (89)
**Laboratory values**	
NT‐proBNP (pg/ml)	1331 [683–2531]
eGFR (ml/min*1.73 m^2^)	55.0 [39.5–77.0]
**Echocardiography**	
LV ejection fraction (%)	54 ± 7
Mitral septal *e*′ velocity (cm/s)	7.7 [6.0–9.6]
Mitral lateral septal *e*′ velocity (cm/s)	6.5 [5.7–7.9]
Mean septal/lateral *e*′ velocity (cm/s)	7.2 [6.1–8.6]
LV *E*/*e*′	12.2 [8.9–16.3]
TAPSE (mm)	20.3 ± 4.5
LA volume index (ml/m^2^)	43 ± 13

*Note*: Quantitative data are presented as mean ± SD or median with interquartile ranges. Qualitative data are presented as *n* (%).

Abbreviations: ACEI, angiotensin converting enzyme inhibitor; ARB, angiotensin II receptor blocker; COPD, chronic obstructive pulmonary disease; eGFR, estimated glomerular filtration rate; LA, left atrium; LV, left ventricle; MRA, mineral corticoid receptor antagonist; NYHA, New York Heart Association; TAPSE, tricuspid annular plane systolic excursion.

### 3.2 Associations between EAT and clinical characteristics

The associations between total EAT and clinical characteristics are shown in Table S1. Male sex and BMI, as well as systolic blood pressure, previous myocardial infarction, diabetes mellitus, NYHA class, NTproBNP, eGFR, and HbA1c were all significantly associated with total EAT. Contrastingly, atrial fibrillation and age were not associated with EAT.

### 3.3 EAT and RV‐structure and function

The associations between the location of EAT and adjacent myocardial structure and function are depicted in Table 2. EAT around the RV was strongly associated with an increased RV mass (*β* = 0.60, *p* < .001), and remained significantly associated with RV mass after adjusting for total EAT, sex, NTproBNP, eGFR, and HbA1c (*β* = 0.21, *p* = .02). The association between RV‐EAT and RV mass was comparable between patients with a BMI above and below 25 kg/m^2^ (*β* = 0.56, *p* = .009 and *β* = 0.59, *p* < .001). RV‐EAT was not independently associated with other indices of RV‐structure and function. RV‐EAT was not associated with LV or atrial structure and function in multivariable analysis (Table S2).

**TABLE 2:** Associations between EAT location and myocardial structure on CMR

	HF patients			
	*RV EAT*			
	Unadjusted		Adjusted	
Right ventricular function and structure	*β*	*p*‐value	*β*	*p*‐value
RV mass (g/m^2^)	0.60	<.001	0.21	.02
RV ejection fraction (%)	−0.17	.09	−0.06	.4
RV end‐diastolic volume (ml/m^2^)	0.19	.05	0.06	.4
RV end‐systolic volume (ml/m^2^)	0.22	.02	0.03	.6
RV global longitudinal strain (%)	−0.11	.28	0.05	.5

	*LV EAT*			
	Unadjusted		Adjusted	
Left ventricular function and structure	*β*	*p*‐value	*β*	*p*‐value
LV mass (g/m^2^)	0.24	.02	0.12	.1
LV ejection fraction (%)	−0.31	.002	−0.12	.11
LV end‐diastolic volume (ml/m^2^)	0.47	<.001	0.24	.001
LV end‐systolic volume (ml/m^2^)	0.43	<.001	0.22	.002
LV global longitudinal strain (%)	−0.29	.003	−0.1	.2
LV global circumferential strain (%)	−0.24	.01	−0.04	.5

	*Atrial EAT*			
	Unadjusted		Adjusted	
Right and left atrial structure	*β*	*p*‐value	*β*	*p*‐value
LA volume index (ml/m^2^)	0.16	.12	−0.03	.7
RA volume index (ml/m^2^)	0.13	.21	−0.09	.4

*Note*: Associations between location of EAT and adjacent myocardial structure and function. Shown are unadjusted values as well as adjusted for total EAT, sex, NTproBNP, eGFR, and HbA1c.

Abbreviations: EAT, epicardial adipose tissue; LA, left atrium; LV, left ventricle; RA, right atrium; RV, right ventricle.

### 3.4 EAT and LV‐structure and function

EAT around the LV was associated with LV mass in univariable analysis (*β* = 0.24, *p* = .02), but this relation was lost in multivariable analysis (*β* = 0.12, *p* = .1). Instead, a more robust association was detected between LV‐EAT and increased LV volumes (LV end‐diastolic volume *β* = 0.24, *p* = .001) and LV end‐systolic volume *β* = 0.22, *p* = .002 in multivariable analysis. Upon echocardiographic analysis, EAT around the LV was inversely related to lateral *e*′ velocity (*β* = −0.25, *p* = .02) but not with septal *e*′ velocity (*β* = 0.15, *p* = .19) (data not shown). LV‐EAT was not independently associated with right‐ventricular or atrial structure and function (Table S2).

### 3.5 Association between atrial EAT and atrial fibrillation

Atrial EAT was not associated with atrial volumes in the total population. However, HF patients with a history of atrial fibrillation had higher atrial EAT volumes, compared to patients without a history of atrial fibrillation (31 ml/m^2^ vs. 26 ml/m^2^, *p* = .04), despite having comparable BMI (29 kg/m^2^ and 30 kg/m^2^, *p* = .3). LV and RV EAT volumes were not significantly different between patients with atrial fibrillation and those without atrial fibrillation (Figure S1).

## 4 DISCUSSION

In the present study we demonstrate that regional EAT is associated with alterations in adjacent cardiac function and structure. In addition, the associations between local EAT and neighboring myocardial structure and function were independent of global measures of adiposity. Lastly, HF patients with a history of atrial fibrillation had an increase in atrial EAT compared to HF patients without a history of atrial fibrillation, despite having similar ventricular EAT and BMI. These data are novel, and to our knowledge this is the first study to separately quantify EAT volumes around the LV, RV and atria in HFmrEF/HFpEF patients and show that local EAT is involved in the pathophysiology of HFmrEF and HFpEF.

The exact mechanisms by which EAT accumulation is associated with cardiac conduction, function, and structure are currently being unraveled. Nalliah et al. demonstrated that local‐, but not global, adiposity was related to conduction abnormalities and myocardial fibrosis in the right atrium.^9^ Our finding that local EAT is associated with changes in the adjacent myocardial structure, independent of global measures of adiposity, is in line with the study by Nalliah et al. Furthermore, none of the LV function and structure indices were associated with RV‐EAT and vice versa, suggesting that local‐, rather than global EAT is related to myocardial structure. In the same study by Nalliah et al., histological slices of the right atrial appendage showed clear infiltration of EAT into the underlying myocardium and this was associated with local conduction disturbances.^9^ It is therefore plausible that EAT infiltration is also the mechanism by which local EAT affects the adjacent myocardial structure and function in HFpEF and HFmrEF. Hypothetically, the RV and atria may be more susceptible to EAT infiltration compared to the LV, as they are much thinner. For instance, it was recently observed that increased EAT was associated with higher RV filling pressures at rest, but not higher with LV filling pressures.^7^ Our observation that EAT seems more strongly related to RV hypertrophy, than to LV hypertrophy, supports this hypothesis. However, these statements are speculative and further investigation is needed to assess whether RV structure is indeed more sensitive for EAT accumulation compared to LV structure.

Atrial fibrillation is often seen in patients with HFpEF and is associated with increased mortality.^18^, ^19^ Gaining insight in the pathophysiology of atrial fibrillation in HFpEF patients is therefore paramount. Our finding that patients with atrial fibrillation have increased atrial EAT volume, but not ventricular EAT volume, supports the accumulating evidence that local atrial EAT plays a role in atrial fibrillation pathophysiology. For the atria, EAT accumulation may lead to electroanatomical and structural remodeling, ultimately resulting in atrial fibrillation.^9^, ^20^ Moreover, recent data suggest that atrial EAT may have distinct characteristics compared to ventricular EAT, but this needs further investigation.^21^

### 4.1 Study limitations

The present study has several potential limitations. First, when measuring EAT, we could not entirely rule out the presence of pericardial effusion. However, the T1 values of the EAT depots corresponded with the T1 times of subcutaneous fat, and not water. In addition, recent echocardiography was checked for pericardial effusion, which was not observed in these patients, therefore minimizing the chance of overestimation of EAT. Second, due to the cross‐sectional, retrospective nature of the study, we could not explore direct casual relations between location of EAT, myocardial function and structure. Third, to control for global visceral adiposity, the gold‐standard would be to measure abdominal visceral adipose tissue using MR imaging. However, we did not have abdominal MR images at our disposal and chose to use BMI as a surrogate for global adiposity instead.

### 4.2 Clinical implications

Our study has shown that regional‐, rather than global, EAT is involved in the pathophysiology of patients with HFpEF and HFmrEF. Therefore, precise localization of EAT is important and can easily be done using CMR imaging. Furthermore, these data give a greater understanding of the mechanism linking EAT to HFpEF/HFmrEF pathophysiology and hopefully lead to the development of new therapies for these HF patients.

## 5 CONCLUSIONS

Regional EAT is strongly associated with local cardiac structure and function in HF patients with LVEF >40%. These data support the hypothesis that regional EAT is involved in the pathophysiology of HF with LVEF >40%.

## Supporting information


**Figure S1** Bar graphs comparing different epicardial adipose tissue (EAT) volumes in heart failure patients with or without atrial fibrillation. (*p < .05). Volumes are indexed for body surface area.

**Table S1** Associations between total EAT and clinical characteristics.

**Table S2** Associations between EAT location and opposite myocardial structure on CMR.

## Data Availability

Data of this research are not shared.
