# Supplementary figures and images for: Importance of epicardial adipose tissue localization using cardiac magnetic resonance imaging in patients with heart failure with mid‐range and preserved ejection fraction
Source: Clin Cardiol. 2021 Jun 4;44(7):987–93. doi: 10.1002/clc.23644 (PMC8259147; doi:10.1002/clc.23644)

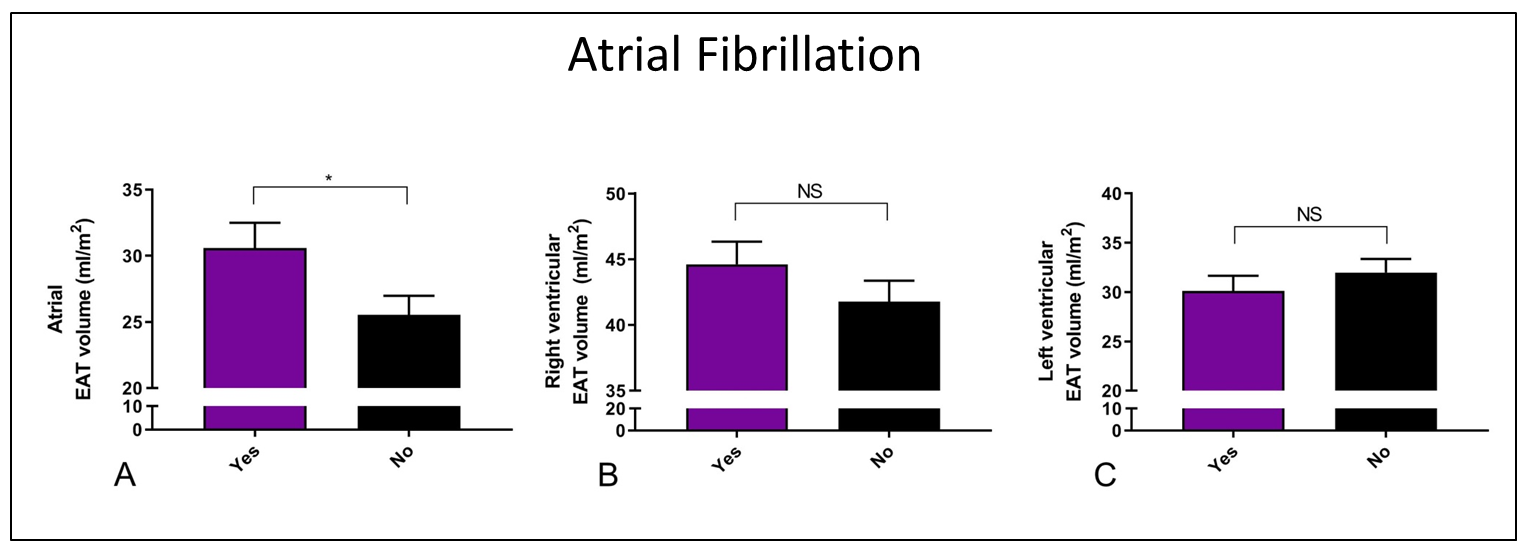

Supplement: Supplementary file 1 — Figure S1 Bar graphs comparing different epicardial adipose tissue (EAT) volumes in heart failure patients with or without atrial fibrillation. (*p < .05). Volumes are indexed for body surface area. [file CLC-44-987-s001.tif]
