# Supplementary material for: Importance of epicardial adipose tissue localization using cardiac magnetic resonance imaging in patients with heart failure with mid‐range and preserved ejection fraction
Source: Clin Cardiol. 2021 Jun 4;44(7):987–93. doi: 10.1002/clc.23644 (PMC8259147; doi:10.1002/clc.23644)
Supplement: Supplementary file 2 — Table S1 Associations between total EAT and clinical characteristics. [file CLC-44-987-s002.docx]

**Supplementary table 1.** Associations between total EAT and clinical characteristics

|  | **β with total EAT** | **p** |
| --- | --- | --- |
|  |  |  |
| Age | 0.004 | 0.9 |
| Sex | 0.26 | **0.01** |
| Body mass index | 0.22 | **0.03** |
| ***Vital signs*** |  |  |
| Systolic blood pressure | -0.22 | **0.04** |
| Diastolic blood pressure | -0.13 | 0.2 |
| Heart rate | -0.06 | 0.6 |
| ***Comorbidities*** |  |  |
| Hypertension | 0.07 | 0.5 |
| Atrial fibrillation | 0.13 | 0.2 |
| Myocardial infarction | 0.20 | **0.049** |
| Diabetes mellitus | 0.30 | **0.003** |
| COPD | 0.09 | 0.4 |
| NYHA class | 0.25 | **0.02** |
| ***Laboratory values*** |  |  |
| LnNT-proBNP | 0.25 | **0.02** |
| LneGFR | -0.36 | **<0.001** |
| LnHbA1c | 0.34 | **0.005** |
