# Supplementary material for: Importance of epicardial adipose tissue localization using cardiac magnetic resonance imaging in patients with heart failure with mid‐range and preserved ejection fraction
Source: Clin Cardiol. 2021 Jun 4;44(7):987–93. doi: 10.1002/clc.23644 (PMC8259147; doi:10.1002/clc.23644)
Supplement: Supplementary file 3 — Table S2 Associations between EAT location and opposite myocardial structure on CMR. [file CLC-44-987-s003.docx]

**Supplementary table 2.** Associations between EAT location and opposite myocardial structure on CMR

|  | **HF patients** |  |
| --- | --- | --- |
|  | **LV EAT** |  |
|  | **Adjusted** |  |
| **Right ventricular function and structure** | **β** | **p-value** |
| RV mass (g/m2) | 0.15 | 0.1 |
| RV ejection fraction (%) | 0.13 | 0.07 |
| RV end-diastolic volume (ml/m2) | 0.01 | 0.8 |
| RV end-systoIic volume (ml/m2) | -0.07 | 0.3 |
| RV global longitudinal strain (%) | 0.11 | 0.1 |
|  |  |  |
|  | **RV EAT** |  |
|  | **Adjusted** |  |
| **Left ventricular function and structure** | **β** | **p-value** |
| LV mass (g/m2) | 0.06 | 0.4 |
| LV ejection fraction (%) | -0.04 | 0.6 |
| LV end-diastolic volume (ml/m2) | 0.02 | 0.8 |
| LV end-systolic volume (ml/m2) | 0.03 | 0.7 |
| LV global longitudinal strain (%) | -0.03 | 0.7 |
| LV global circumferential strain (%) | -0.02 | 0.8 |
|  |  |  |
|  | **Ventricular EAT** |  |
|  | **Adjusted** |  |
| **Right and left atrial structure** | **β** | **p-value** |
| LA volume index (ml/m2) | 0.02 | 0.8 |
| RA volume index (ml/m2) | 0.06 | 0.3 |

Assocations between location of EAT and opposite myocardial structure and function. Shown are values adjusted for total EAT, sex, NTproBNP, eGFR and HbA1c. EAT=epicardial adipose tissue, LA=left atrium, LV=left ventricle, RA=right atrium, RV=right ventricle,
